# Multiscale Cumulative Residual Dispersion Entropy with Applications to Cardiovascular Signals

**DOI:** 10.3390/e25111562

**Published:** 2023-11-20

**Authors:** Youngjun Kim, Young-Seok Choi

**Affiliations:** Department of Electronics and Communications Engineering, Kwangwoon University, Seoul 01897, Republic of Korea

**Keywords:** electrocardiogram, heart rate variability, R-R interval, cumulative residual dispersion entropy, complexity

## Abstract

Heart rate variability (HRV) is used as an index reflecting the adaptability of the autonomic nervous system to external stimuli and can be used to detect various heart diseases. Since HRVs are the time series signal with nonlinear property, entropy has been an attractive analysis method. Among the various entropy methods, dispersion entropy (DE) has been preferred due to its ability to quantify the time series’ underlying complexity with low computational cost. However, the order between patterns is not considered in the probability distribution of dispersion patterns for computing the DE value. Here, a multiscale cumulative residual dispersion entropy (MCRDE), which employs a cumulative residual entropy and DE estimation in multiple temporal scales, is presented. Thus, a generalized and fast estimation of complexity in temporal structures is inherited in the proposed MCRDE. To verify the performance of the proposed MCRDE, the complexity of inter-beat interval obtained from ECG signals of congestive heart failure (CHF), atrial fibrillation (AF), and the healthy group was compared. The experimental results show that MCRDE is more capable of quantifying physiological conditions than preceding multiscale entropy methods in that MCRDE achieves more statistically significant cases in terms of *p*-value from the Mann–Whitney test.

## 1. Introduction

The physiological system is regulated by systems interacting across multiple spatial and temporal scales. Such systems generate complex variations with information related to dynamic systems [1]. The complexity of physiological systems has a property of dynamic models reflecting the ability to respond and adapt to the ever-changing environment. Thus, quantifying a system’s complexity is a prospective tool for analyzing physiological systems with non-static, nonlinear, and complex behaviors [2,3,4,5]. Such complexity analysis of physiological signals can help to extract primary information about the dynamic relationship of systems in changes related to human statuses, such as aging, emotions, and diseases. Moreover, it becomes essential to quantify physiological signals in clinical diagnosis and prognosis cases for medical devices and healthcare, which have recently received increasing attention.

Entropy has been broadly used as a measure to prove the existence of deterministic chaos from data [6,7]. Richman et al. devised sample entropy (SampEn) which analyzes the degree of self-similarity of the signals [8]. SampEn has been utilized in analyzing various types of signals [9,10]. Despite SampEn’s capability, it is vulnerable to short-length signals and is not feasible for real-time applications due to computational complexity, especially for long signals. Another widely used entropy has been permutation entropy (PE), which captures the order relations between values of a time series and extracts a probability distribution of the ordinal patterns. Although PE is computationally simple and fast [11,12], it does not consider the amplitude of information, such as the mean value of amplitudes and differences between amplitudes values.

As an alternative entropy measure, dispersion entropy (DE), which uses symbolic patterns and Shannon entropy to quantify the uncertainty of time series, has been introduced [13]. DE generates symbolic patterns, named dispersion patterns, transforming an original time series into a new signal with only a few constituents. As a result, some specific information may be lost, but certain invariant and robust features may be preserved [14,15,16]. Unlike PE, DE does not need to calculate every distance between two composite delay vectors consisting of embedding dimensions *m* and *m* + 1, so DE gains a lower complexity cost. In addition, DE is more capable of capturing features of changing amplitude, frequency, and bandwidth of time series. Thus, DE can be a more suitable method than SampEn and PE for real-time processing applications such as medical diagnosis.

Despite these strengths of DE, it also has drawbacks. DE does not consider the order of each pattern in the probability distribution of the dispersion pattern. For example, suppose there are four dispersion patterns for any two signals. The probability of each dispersion pattern of one signal is $P_{1}=\left\{0.4,\;0.3,\;0.2,\;0.1\right\}$ and the other one is $P_{2}=\{0.1,\;0.2,\;0.3,\;0.4\}$. The DE values for these two distributions are equal because each entropy value is calculated with only each probability without considering the order of patterns. However, those two probability distributions are different, so the ability to distinguish between the signals forming those two distributions is not sufficient. This could make distinguishing between a biological signal with a high distribution of dispersion patterns corresponding to a low amplitude and one with a high distribution of dispersion patterns relating to a high amplitude challenging. As a result, reliable clinical diagnosis may be difficult. A cumulative residual entropy (CRE) has been used to solve this problem [17]. CRE uses a cumulative distribution function instead of a probability density function to identify the information for continuous variables. The probability distribution function in CRE results in more generality and universal properties than the conventional Shannon entropy [17,18]. CRE has been utilized in a variety of applications in this regard, including image signal processing and pattern recognition [16,17,19,20]. Several studies have shown that considering the order of the patterns improves the analysis of physiological and clinical signals [21,22]. However, the entropy methods mentioned above measure the irregularity of the time series, yielding that it may fail to capture the complexity of the time series. To solve this issue, Costa et al. [23,24] have developed a multiscale entropy analysis that characterizes the complexity of a time series over multiple scales of time. This multiscale entropy analysis has effectively represented the dynamical characteristics of an underlying time series.

Here, a multiscale cumulative residual dispersion entropy (MCRDE), which computes the cumulative residual dispersion entropy (CRDE) over multiple temporal scales, is presented. Combining three entropy algorithms for the first time, the suggested method inherits the benefits of DE, CRE, and multiscale entropy. As a result, the proposed MCRDE improves the capability for quantifying the temporal dynamics of the underlying time series. To validate the capability of the proposed MCRDE, we first compare the performance of MCRDE with the conventional multiscale SampEn (MSE) and multiscale DE (MDE) using synthetic signals, i.e., the white Gaussian noise (WGN) and 1/f noise. Next, the proposed MCRDE is applied to inter-beat (RR) intervals extracted from the ECG signals of congestive heart failure (CHF) patients, atrial fibrillation (AF) patients, and healthy subjects. Through experiments, using public ECG datasets, the proposed MCRDE leads to an improved capability for quantifying physiological status compared to the conventional multiscale entropy methods regardless of the length of inter-beat intervals.

The remainder of this paper is organized as follows: DE, CRE, and the proposed MCRDE are introduced in Section 2. In Section 3, the results using synthetic signal and ECG datasets are presented to verify the effectiveness of the proposed MCRDE. Then, discussions for the results are described in Section 4. Finally, Section 5 presents the conclusions.

## 2. Materials and Methods

### 2.1. Dispersion Entropy (DE)

**Figure 1:**
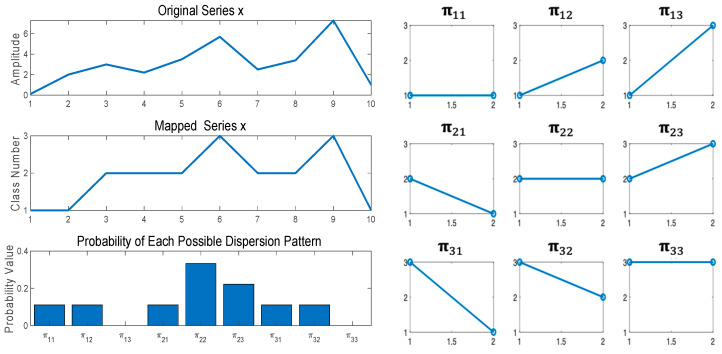
Examples of the DE algorithm using series $x=\left\{0.1\;2\;3\;2.2\;3.5\;5.7\;2.5\;3.4\;7.3\;1\right\}$ with c=3, m=2, τ=1.

Assume we have a signal of length *N:* $x=\{x_{1},\;x_{2},\;\cdots ,\;x_{N}\}$; DE algorithm is composed of the following four steps as follows [13]:

(1) First, $x_{i}(i=1,\;2,\cdots ,\;N)$ are mapped to *c* classes labeled from 1 to *c*. In the mapping process, there are various linear and nonlinear mapping techniques. Although the linear mapping algorithm is computationally fast, if the maximum or minimum values of a signal are much larger or smaller than the mean or median value of the signal, the majority of $x_{i}$ is biased only toward few classes. Here, the normal cumulative distribution function (NCDF) for mapping x into $y=\{y_{1},\;y_{2},\;\cdots ,\;y_{N}\}$ from 0 to 1 is used. Each embedding vector $u_{i}^{m,c}$ is made with embedding dimension *m* and time delay *d* according to $u_{i}^{m,c}=\left\{u_{i}^{c},\;u_{i+d}^{c},\;\cdots ,u_{i+\left(m-1\right)d}^{c}\right\},\;i=1,\;2,\cdots ,\;N-\left(m-1\right)d$. Each time series $u_{i}^{m,c}$ is mapped to a dispersion pattern $\pi_{v_{0},{\;v}_{1},\;\cdots ,v_{m-1}\;},$ where $u_{i}^{c}=v_{0},u_{i+d}^{c}=v_{1},\;\cdots ,\;u_{i+\left(m-1\right)d}^{c}=v_{m-1}$. The number of available dispersion patterns can be assigned to each vector $u_{i}^{m,c}$ which is equal to $c^{m}$ because the signal is made up of *m* members and each can be one of the integers from 1 to *c*.
(2) For each of $c^{m}$ possible dispersion patterns $\pi_{v_{0},{\;v}_{1},\;\cdots ,v_{m-1}\;},$ relative frequency is computed as follows:
  (1) $$p\left(\pi_{v_{0},\;\cdots ,v_{m-1}\;}\right)=\frac{\#\{i|i\leq N-\left(m-1\right)d,\;u_{i}^{m,c}\;has\;type\;\pi_{v_{0},\;\cdots ,v_{m-1}\;}\}\;}{N-\left(m-1\right)d}$$
  where # denotes cardinality. In fact, $p\left(\pi_{v_{0},\;\cdots ,v_{m-1}\;}\right)$ denotes the number of dispersion patterns $\pi_{v_{0},{\;v}_{1},\;\cdots ,v_{m-1}}$ that are assigned to $u_{i}^{m,c}$ divided by the whole number of embedded signals with embedding dimension *m*.
(3) Lastly, DE is obtained using the Shannon entropy approach [25] as follows:
  (2) $$DE\left(x,m,c,d\right)=-\sum_{\pi =1}^{c^{m}}p\left(\pi_{v_{0},\;\cdots ,v_{m-1}\;}\right)\cdot \ln\left(p\left(\pi_{v_{0},\;\cdots ,v_{m-1}\;}\right)\right)\;$$
  For example, x={0.1 2 3 2.2 3.5 5.7 2.5 3.4 7.3 1} is considered and shown on the top left of Figure 1. DE of x with *d* = 1, *m* = 2, and *c* = 3 is computed in Table 1 and Figure 1. Table 1 shows the dispersion patterns and their probability. Figure 1 shows the time series x, classified series z, potential dispersion patterns and probability of each potential dispersion pattern.

Next, a window with length 2, which is an embedding dimension, is moved along the time series and the number of each of the corresponding dispersion patterns is counted. Finally, using Equation (2), DE value of x is calculated as $DE(x,2,\;3,\;1)=-\sum p\left(\pi \right)\ln\left(p\left(\pi \right)\right)=1.0297$. It is noted that if all potential patterns occur equally, the value of DE will be the largest. On the other hand, if only certain patterns happen regularly, the value of DE will be the smallest.

### 2.2. Cumulative Residual Entropy (CRE)

Cumulative residual entropy (CRE) is more applicable and generable than traditional Shannon entropy since it is plausible for continuous distributions [17,18]. For a given random vector $x=\{x_{1},\;x_{2},\;\cdots ,\;x_{N}\}\in R^{N}$, CRE is obtained as follows:

(3) $$CRE\left(x\right)=-\int_{R_{+}^{N}}^{\;}P\left(\left|x\right|>\lambda \right)\log P\left(\left|x\right|>\lambda \right)d\lambda$$

where $\lambda =(\lambda_{1},\lambda_{2},\cdots ,\lambda_{N})$, $\left|x\right|>\lambda$ represents $|x_{i}|>\lambda_{i}$ and $R_{+}^{N}=\left(x_{i}\in R^{N};x_{i}\geq 0\right).$

Next, CRE computation is applicable for a discrete version. For independent and identical distributed discrete random variables, $F\left(x\right)$ is the cumulative density function and $F_{n}\left(x\right)=\frac{1}{n}\sum_{i=1}^{n}I_{\left\{x\geq x_{i}\right\}}$ is empirical distribution function corresponding to each random variable, where $I_{\left\{x\geq x_{i}\right\}}$ is the indicative function. CRE is obtained using

(4) $$\begin{array}{lll} \mathrm{CRE}(x) & = & -\int_{0}^{\infty }\left(1-F_{n}\left(x\right)\right)\log\left(1-F_{n}\left(x\right)\right)dx\\ & = & -\frac{1}{n}\sum_{i=1}^{n-1}\frac{n}{n-i}log\frac{n}{n-i}(x_{i+1}-x_{i}) \end{array}$$

Here, it is assumed that x is order statistics. In addition, the empirical distribution function is obtained using

(5) $$F_{n}\left(x\right)=\left\{\begin{array}{c} \begin{array}{c} 0,\;\;\;\;\;\;\;\;\;\;\;\;\;\;\;\;x<x_{1}^{*}\\ \;\;\;\frac{k}{n},\;\;\;\;\;x_{k}^{*}\;\leq x<x_{k+1}^{*} \end{array}\\ 1,\;\;\;\;\;\;\;\;\;\;\;\;\;\;x\geq \;x_{N}^{*} \end{array}\right.$$

where $x_{1}^{*}\;\leq x_{2}^{*}\leq \cdots \leq x_{N}^{*}$ are ascending order statistics.

CRE possesses the following properties: (1) Although both continuous and discrete variables have valid definitions, estimating the empirical distribution for differential entropy of continuous variables is difficult. (2) CRE has nonnegative values. (3) CRE eventually converges.

### 2.3. Cumulative Residual Dispersion Entropy (CRDE)

For an *N* possible dispersion patterns, the entropy of s is obtained with $-\sum_{k=1}^{N}P\{s_{k}\}\cdot log(P\left\{s_{k}\right\})$ based on classical Shannon entropy [25]. Here, $P\{s_{k}\}$ means the probability of occurrence of the dispersion pattern $s_{k}$. Assume that two distinct signals have four dispersion patterns $s=\left\{s_{1},\;s_{2},s_{3},\;s_{4}\right\}.$ As mentioned in the introduction, DE does not consider the order between each dispersion pattern while calculating the entropy value.

This study addresses the above shortcoming of DE by integrating CRE, thus yielding cumulative residual dispersion entropy (CRDE). The proposed CRDE is computed as follows: First, the probability distribution for possible dispersion patterns was computed as DE. Second, we calculate the cumulative density function for the probability distribution of dispersion patterns. Finally, CRDE is obtained with

(6) $$CRDE\left(x\right)=-\sum_{j=1}^{K}\left(1-\sum_{i=1}^{j}P\left\{s_{i}\right\}\right)\cdot log\left(1-\sum_{i=1}^{j}P\left\{s_{i}\right\}\right)\;$$

where *K* is the total number of dispersion patterns, and $P\left\{s_{i}\right\}$ is the probability of occurrence of a dispersion pattern $s_{i}$. Since forming the dispersion pattern is the same as DE, CRDE can maintain the property of DE that is sensitive to amplitude values and bandwidth of time series.

### 2.4. Multiscale Analysis of CRDE

To make CRDE applicable to the multiscale analysis of time series, a coarse-graining procedure is integrated to generate multiple sets of time series with different time scales. For a given original time series $x=\{x_{1},\;x_{2},\;\cdots ,\;x_{N}\}$ of length *N* is divided into non-overlapping windows according to the time scale factor *s*. Then, a consecutive coarse-grained time series $y^{s}=\{y_{1}^{s},\;y_{2}^{s},\;\cdots ,\;y_{N/s}^{s}\}$ is developed, which consist of multiple $y_{j}^{s}$. It is obtained with

(7) $$y_{j}^{s}=\frac{1}{s}\sum_{i=\left(j-1\right)s+1}^{j\cdot s}x_{i},\;\;\;\;\;\;(1\leq j\leq \frac{N}{s})$$

For the scale factor s=1, the coarse-grained time series $y^{1}=\{y_{1}^{1},\;y_{2}^{1},\;\cdots ,\;y_{N}^{1}\}$ is identical with the original time series x. In general, the length of the time series after coarse-graining is equal to *N* of the original time series divided by the scale factor *s*. This multiscale analysis allows the assessment of the dynamic complexity associated with the ability of physiological systems to adapt to changing environments. Finally, we calculate multiscale CRDE (MCRDE) on the coarse-grained time series as follows:

(8) $$MCRDE\left(x,s\right)=CRDE\left(y^{s}\right).$$

### 2.5. Synthetic Data and Real ECG Data

**Figure 2:**
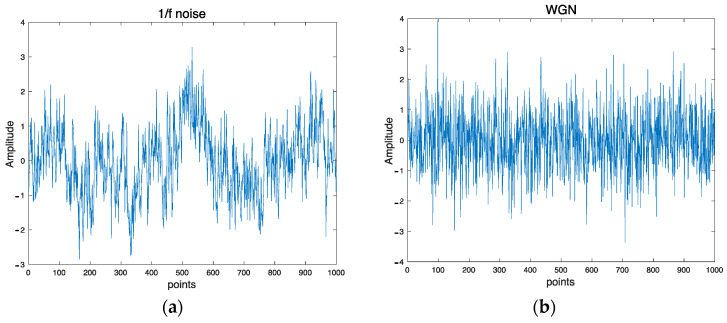
Examples of synthetic signals: (**a**) 1/f noise and (**b**) WGN.

The synthetic data used in this work are 1/f noise and White noise. 1/f noise is also called pink noise. It is one of the most common behaviors of biological systems. This noise possesses a long-range autocorrelation property in which the power spectral density is inversely proportional to the frequency of a signal. In contrast with White noise, it has a constant power spectral density at different frequencies. Figure 2a,b depict examples of 1/f noise and WGN, respectively.

**Figure 3:**
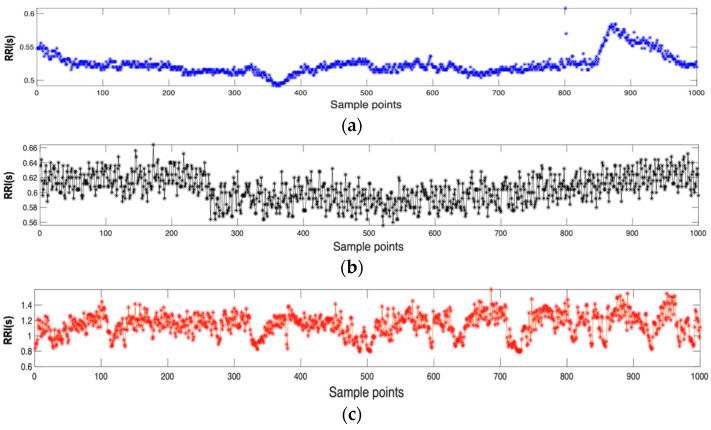
Representative inter-beat (RR) intervals extracted from the electrocardiogram (ECG) dataset: (**a**) congestive heart failure (CHF) group, (**b**) atrial fibrillation (AF) group, (**c**) healthy group.

Real ECG datasets, i.e., BIDMC CHF, MIT-BIH AF, and Fantasia are obtained in PhysioNet [26]. Specifically, BIMD CHF contains ECG records of 15 patients with CHF, consisting of eleven men aged 22–47 years and four women aged 54–63 years. Each recording is approximately 20 h in duration with a sampling frequency of 250 Hz. MIT-BIH AF database includes 25 long-term (10 h) ECG recordings of twenty-three paroxysmal and two persistent. The Fantasia dataset contains 20 healthy subjects aged 21–34 years and 20 elderly subjects aged 68–85 years. Each recording is approximately 2 h in duration and sampled at 250 Hz. RR intervals with lengths of *N* = 1000 were randomly extracted from ECG recordings. Recording an ECG signal typically involves placing electrodes on the skin at specific locations to capture the electrical activity of the heart. Then, the ECG lead that gives the clearest R waves is chosen, followed by the detection of QRS waves. The R and QRS waves are extracted using the Pan-Tompkins algorithm [27]. Then, the RR interval on the ECG signal is obtained as the time between consecutive R waves, which are the prominent upward spikes seen on an ECG trace. The representative RR interval time series of CHF patient, AF patient, and healthy subject are shown in Figure 3a–c. For analyzing ECG signals, MATLAB 2020b version was used.

### 2.6. Statical Analysis Method

We performed the Mann–Whitney U test, also known as the Wilcoxon rank-sum test, to verify whether a distinction using the proposed MCRDE between different groups is statistically significant. The Mann–Whitney U test is a non-parametric statistical test that is used to determine whether there is a difference between two independent samples. It is often used when the data are not normally distributed or when the sample sizes are small. In this test, the null hypothesis was that the two groups are indifferent, and the significance probability p-value was the probability of an observed result assuming that the null hypothesis is true. Generally, if the p-value is less than 0.05 or 5%, the results are considered “statistically significant”. This implies that the observed data or results are unlikely to be due to chance, suggesting a high likelihood of a meaningful difference between the entropy values of different groups.

## 3. Results

### 3.1. Simulations Using Synthetic Data

In order to compare the performance of the proposed MCRDE to that of the conventional MDE, the simulations using two synthetic signals, i.e., 1/f noise and WGN were conducted. In this simulation, the predefined parameters for DE as the number of classes c = 3 and the embedding dimension m = 3 were used.

**Figure 4:**
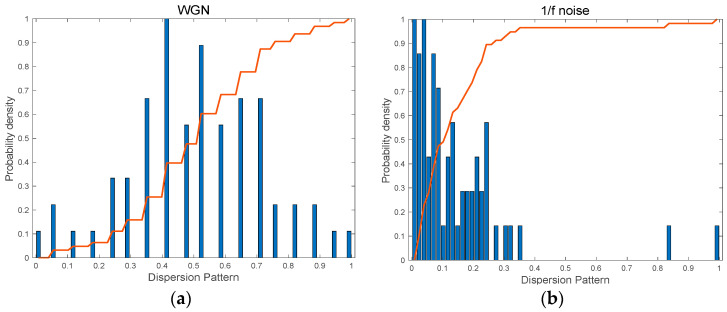
The histogram for possible dispersion patterns and cumulative distribution curves (the solid red line) for synthetic signals: (**a**) WGN, N = 1000 and (**b**) 1/f noise, N = 1000.

Figure 4 shows the probability density function (PDF) of possible dispersion patterns which are presented with normalized values and corresponding cumulative distribution curves of the probability distribution for the possible dispersion patterns. The histogram of the dispersion pattern for WGN is roughly a normal distribution shown in Figure 4a. However, 1/f noise has a left-skewed distribution. As can be seen, 1/f noise has a steeper accumulation rate, resulting in relatively higher complexity than WGN.

**Figure 5:**
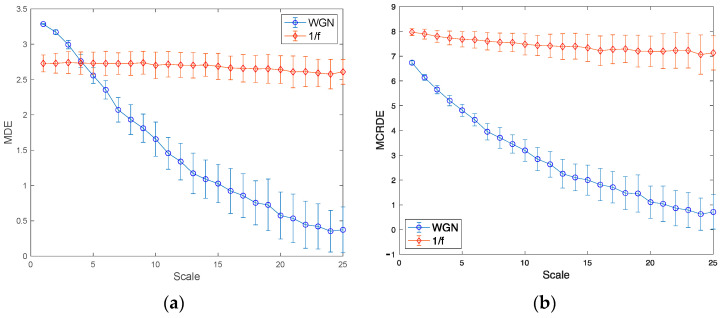
Entropy values for synthetic signals: (**a**) results of MDE for N = 1000; (**b**) results of MCRDE for N = 1000; scale range of 1–25 are used, and the value at each scale represents a mean ± standard deviation.

Figure 5a,b demonstrate the entropy values of MDE and MCRDE consisted of 50 different 1/f noise and WGN with the length of N = 1000, respectively. It has been known that 1/f noise has a higher complexity than WGN [26,27]. The results of MDE in Figure 5a show two folds: First, at small scale factors less than 5, MDE values of WGN are higher than those of 1/f noise. Second, as the scale factor increases, MDE values of 1/f noise remain nearly constant, while MDE values of WGN monotonically decrease. The results of MCRDE in Figure 5b shows that MCRDE values of 1/f noise computed remain almost constant. In addition, the entropy values of WGN by MCRDE are less than those of 1/f noise by MCRDE on all scale factors and decrease as the scale factors increase. These results imply that the proposed MCRDE is more capable of discriminating complexity in underlying synthetic signals compared to MDE.

### 3.2. Experimental Results of ECG Dataset

#### 3.2.1. Comparison of Entropy Measures for Distinct Cardiovascular Signals

Using the ECG database, the RR interval time series extracted from the ECG signals were analyzed using MSE, MDE, and the proposed MCRDE. The Mann–Whitney U test was used to verify the statistical difference among the three groups. Here, we set the significance level of the hypothesis test decision to 0.05; thus, statistical significance is accepted in cases of p<0.05.

**Figure 6:**
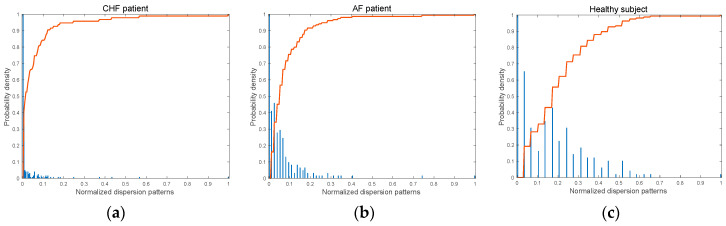
The histogram for possible dispersion patterns and cumulative distribution curves (the solid red line) for RR intervals of three groups: (**a**) CHF patient, (**b**) AF patient, and (**c**) healthy subject.

Figure 6a–c depict the histograms for dispersion patterns and corresponding cumulative distribution curves for the RR interval time series of three groups, respectively. As can be seen, the slope of the cumulative distribution curve decreases in the order of CHF patient, AF patient, and healthy subject. As shown in Figure 6, the variation in RR intervals of CHF patients is the smallest among the three groups. In addition, the occurrence of the dispersion patterns is concentrated on low values, and the slope of the cumulative distribution curve is the highest among the three groups. In the case of AF patients, the RR interval shows more diverse dispersion patterns, and the slope of the cumulative distribution curve is smaller than that of CHF. Lastly, the RR interval of the healthy subject shows the most significant variation among the three groups, implying that more diverse dispersion patterns occur, and thus, its slope of the cumulative distribution curve is the lowest. 

**Figure 7:**
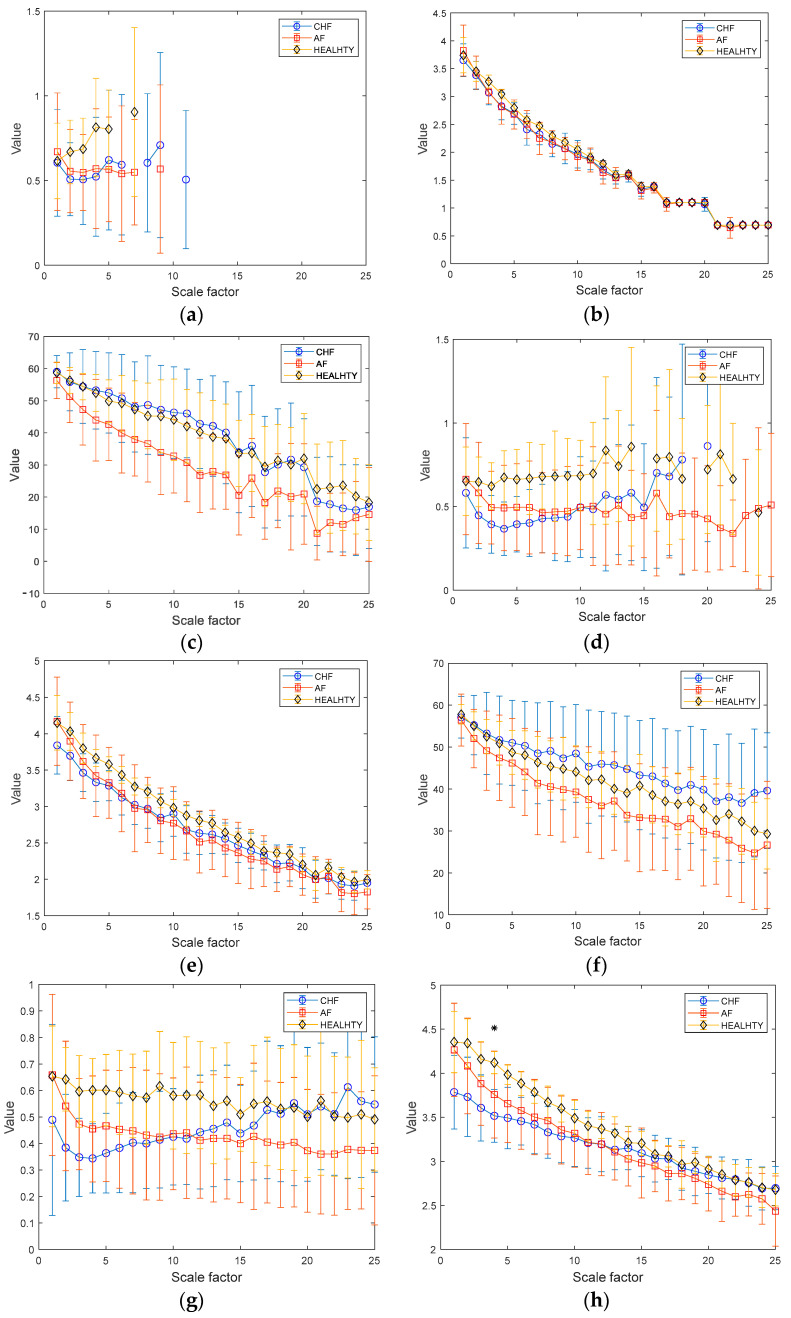

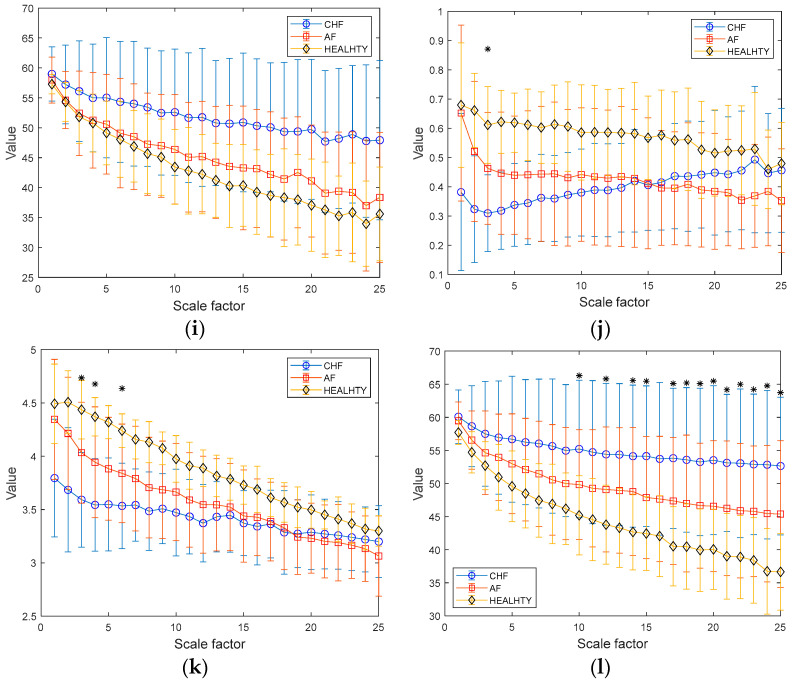
MSE, MDE, and MCRDE results of RR intervals for CHF patients, AF patients and healthy group: (**a**) MSE for N = 100; (**b**) MDE for N = 100; (**c**) MCRDE for N = 100; (**d**) MSE for N = 250; (**e**) MDE for N = 250; (**f**) MCRDE for N = 250; (**g**) MSE for N = 500; (**h**) MDE for N = 500; (**i**) MCRDE for N = 500; (**j**) MSE for N = 100; (**k**) MDE for N = 500; (**l**) MCRDE for N = 500. The scale factor ranges from 1 to 25. The entropy value at each scale factor represents a mean ± standard deviation. The asterisks indicate a significant difference between groups obtained via Mann–Whitney U test (*p* < 0.05).

The results of MSE, MDE, and MCRDE for RR interval time series for lengths of N = 100 and 1000 are shown in Figure 7a–c and Figure 7d–f, respectively. Here, to compare the quantification capability of the complexity of short and relatively sufficient lengths of RR intervals, *N* = 100, 250, 500, and 1000 were chosen. In Figure 7a, MSE values are not defined on most scales, highlighting the limitation of MSE in analyzing short-term RR interval time series. In Figure 7b, the MDE values in the case of *N* = 100 exhibit a decreasing trend as the scale factor increases due to the insufficient length of a coarse-grained RR interval. In addition, distinguishing the complexity of the three groups appears to be complicated.

The results of MSE, MDE, and MCRDE for RR Interval time series for lengths of *N* = 100 and 1000 are shown in Figure 7a–c and Figure 7d–f, respectively. Here, to compare the quantification capability of the complexity of short and relatively sufficient lengths of RR intervals, N = 100, 250, 500, and 1000 were chosen. In Figure 7a, MSE values are not defined on most scales, highlighting the limitation of MSE in analyzing short-term RR interval time series. In Figure 7b, the MDE values in the case of N = 100 exhibit a decreasing trend as the scale factor increases due to the insufficient length of a coarse-grained RR interval. In addition, distinguishing the complexity of the three groups appears to be complicated. On the other hand, as illustrated in Figure 7c, the MCRDE values for RR interval time series of length *N* = 100 are defined across all scales. In addition, MCRDE can capture the complexity difference between the three groups.

Figure 7d shows the results of MSE for RR interval with N=250. Although the MSE computation is available on small scale factors, MSE values are not defined on large scale factors. In Figure 7e, MDE values for RR interval with N=250 show that MDE does not easily reflect the complexity difference between CHF, AF, and healthy groups. Figure 7f exhibits the results of MCRDE. As shown in the figure, MCRDE is not only well defined on all scales but also discriminates the complexity of three groups in order of CHF, AF, and healthy subjects.

Figure 7g–i show results of MSE, MDE, and MCRDE for RR interval with N=500, respectively. In Figure 7g, MSE values are obtained, but it is hard to discriminate the complexity of three groups. In Fiigure 7h, MDE values are not able to differentiate three groups. On the other hand, MCRDE in Figure 7i shows the difference in complexity between the three groups more effectively compared to N=100 and 250.

In Figure 7j, the result of the MSE values is defined across most scale factors when the time series is sufficiently long as N = 1000. The MSE values of the healthy group are distinguishable from other groups. However, the gap between MSE values from CHF and AF patients is inconsistent across the scale factors. It may lead to an incapability to discriminate the complexities between the three groups. In Figure 7e, the MDE value for N = 1000 represents an improved capability for differentiating the entropy values from the three groups compared to the results of MDE for N = 100. Although the results of MDE show more discriminative trends than those of MSE, it is possible to distinguish three groups only at the scale factor s=3, 4, and 6. In Figure 7f, MCRDE results represent a more significant improvement in discriminating the complexities of the three groups. The larger the scale factor, the more apparent the difference in the MCRDE values. Moreover, the statistical analysis also shows that the use of MCRDE leads to a significant difference between the three groups at most scale factors above s=10. 

The cumulative distribution of healthy group reaches one more slowly than other groups, implying a broader dispersion pattern. On the contrary, the cumulative distribution of CHF patients rises to one most rapidly due to the significant skewness of its dispersion pattern. The slower the cumulative distribution reaches one, the lower the MCRDE value is.

#### 3.2.2. Comparison of Entropy Measures for Healthy Young and Elderly Groups

We compared the entropy values for the RR interval time series of healthy young and elderly subjects. We chose the length of RR interval of N = 100, 250, 500, and N = 1000.

**Figure 8:**
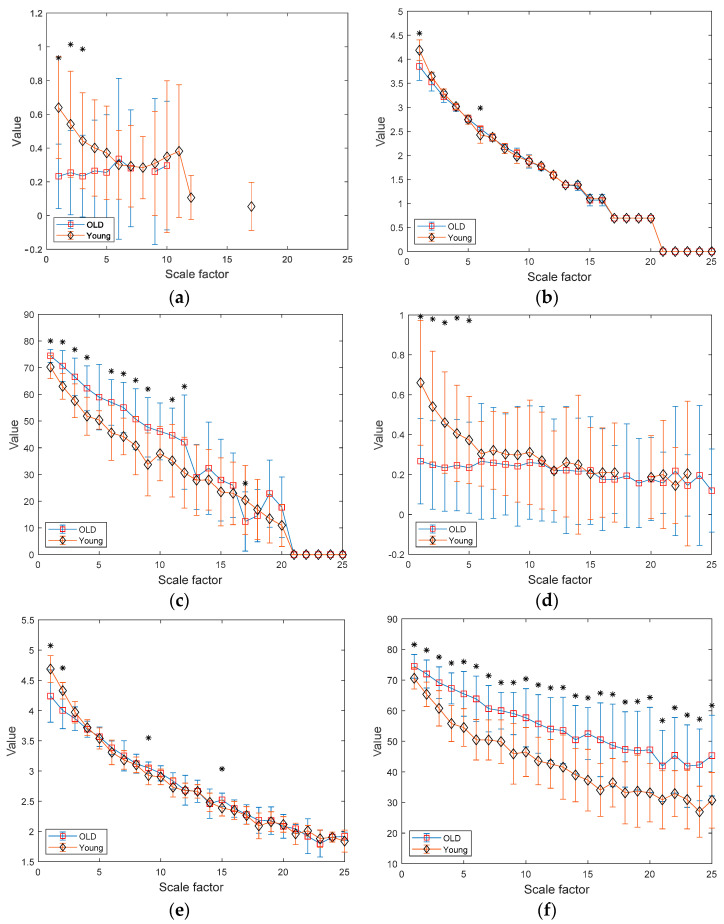

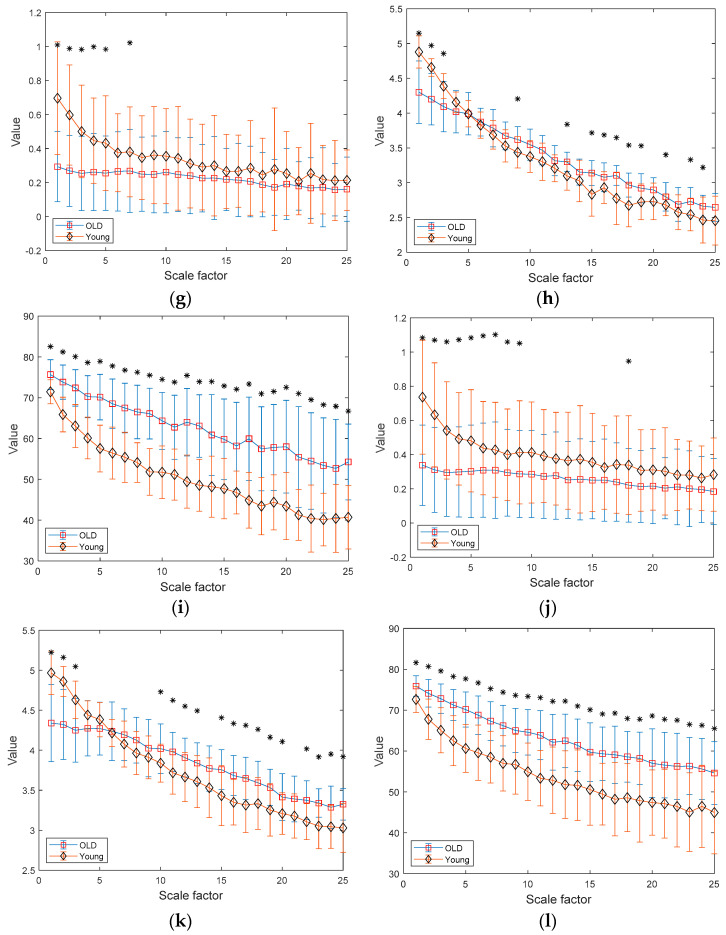
MSE, MDE, and MCRDE results of RR intervals for healthy and old group: (**a**) MSE for *N* = 100; (**b**) MDE for *N* = 100; (**c**) MCRD for N = 100; (**d**) MSE for *N* = 250; (**e**) MDE for *N* = 250; (**f**) MCRDE for *N* = 250; (**g**) MSE for *N* = 500; (**h**) MDE for *N* = 500; (**i**) MCRDE for *N* = 500; (**j**) MSE for *N* = 100; (**k**) MDE for *N* = 500; (**l**) MCRDE for *N* = 500. The scale factor ranges from 1 to 25. The entropy value at each scale factor represents a mean ± standard deviation. The asterisks indicate a significant difference between groups obtained via the Mann–Whitney U test (*p* < 0.05).

Figure 8a–c depict the results of MSE, MDE, and MCRDE for RR interval time series of two groups for N = 100. In Figure 8a, MSE values are not obtained at large scale factors and at small scale factors less than 4, MSE values of two groups are statistically different. In Figure 8b, MDE values of two groups are nearly indistinguishable, thus suffering from statistically discriminating two groups. On the contrary, the MCRDE results in Figure 8c demonstrate its ability to differentiate the complexity between two groups even for short RR interval time series, especially at scale factors less than 13.

In Figure 8d–f, the results of MSE, MDE, and MCRDE for RR interval time series of two groups for N = 250 are shown. In Figure 8d, MSE values at large scale factors such as s=24 and 25 are not defined for young subject, and MSE is capable of differentiating two groups at the scale factors 5 or less. In Figure 8e, MDE values of two groups exhibit similar behavior and it shows statistical difference at the scale factors s=1, 2, 9, and 15. Figure 8f shows that MCRDE is capable of discriminating two groups over all scale factors.

Figure 8g–i shows results of MSE, MDE, and MCRDE for RR interval with N=500, respectively. The MSE results in Figure 8g show that two groups has statistically different complexity at small scale factors. The MDE results in Figure 8h indicate that MDE values of young subjects are higher than those of old subjects at the scale factors s=4 or less, but the opposite trend is shown for the scale factors above 6. In addition, at several scale factors, it is possible to discriminate two groups using MDE values. In Figure 8i, the MCRDE values of old subjects are consistently higher than those of young subjects over all scale factors. Moreover, using MCRDE values leads to significant differentiation between two groups. 

For sufficient long RR interval time series of N = 1000, MSE values are computed over all scale factors and can capture statistical differences at small scale factors, which is shown in Figure 8d. In Figure 8e, MDE values from two groups are differentiable, except for the scale factor between 4–9 and 21. This result implies that MDE is more capable of discriminating two groups than MSE. Finally, the results of MCRDE shown in Figure 8f demonstrate that MCRDE has a superior capability in discriminating the complexity of two groups across all scale factors. Through comparison results in Figure 8, it is clear that MCRDE is suitable for quantifying age-dependent cardiological complexity.

#### 3.2.3. Statistical Analysis of Entropy Measures

In order to evaluate the effectiveness of capturing the difference of complexity in RR interval using MSE, MDE, and MCRDE with various lengths of time series, a statistical analysis was carried out. In addition to empirical comparison in previous sections, the Mann–Whitney U test was utilized to verify whether two healthy groups, i.e., healthy young and elderly groups, can be discriminated. Here, statistical significance is accepted if the *p*-value is less than 0.05 and those *p*-values are marked as gray in Table 2, Table 3, Table 4 and Table 5.

Table 2 depicts the *p*-values in which the MSE values of paired comparison between CHF patients, AF patients, and the healthy group in cases of *N* = 100, 500, and 1000. For N = 100, it is not able to compute MSE values over most scale factors due to the shortage of the length of a coarse-grained RR interval. For *N* = 500 and 1000, it is clear that *p*-value computation is available, and there are increased cases of statistically significant difference. However, it still lacks in distinguishing CHF and AF patients using MSE values.

Table 3 shows the comparison results of MDE. As can be seen, the use of MDE yields an improved capability for distinguishing complexities of different physiological groups than MSE. For *N* = 100, it is possible to compute MDE values for more scale factors and increase the statistically significant cases compared to MSE. In addition, for sufficient long RR intervals as *N* = 500 and 1000, the use of MDE results in a more statistically significant difference than MSE.

In Table 4, the results of MCRDE *N* = 100, 500, and 1000 are shown. For *N* = 100, MCRDE values are computed over all scale factors and yield a much more statistically significant difference, especially between CHF and AF patients as well as AF patients and the healthy group. In addition, the statistical results of MDE in Table 3 show that for sufficient long lengths of RR interval, i.e., *N* = 500 and 1000, the difference utilizing MCRDE values across three groups is statistically significant and better than MSE and MDE. Through comparison between Table 2 and Table 4, the proposed MCRDE shows a superior capability for distinguishing three groups regardless of the length of RR interval.

Lastly, we conducted a statistical analysis to compare the healthy young and healthy elderly subjects. In Table 5, MCRDE exhibits superior discrimination performance with much more statistically significant differences for all lengths of RR interval (*N* = 100, 500, and 1000) compared to conventional MSE and MDE.

## 4. Discussion

This work presents a multiscale version of DE utilizing cumulative distribution with application to the analysis of the cardiovascular signal, i.e., ECG recordings. Various entropy measures play an important role in representing the complexity of neurophysiological signals. Although entropy estimation of neurophysiological signals can to represent the complexity of underlying neural systems to some extent, the relationship between entropy and complexity remains controversial. 

The popular entropy measure, i.e., MSE, provides a solution to address inconsistency with complexity [23]. Due to the capability of MSE, it has been widely used in diverse applications including biomedical environments [28,29,30,31,32,33]. Unlike other applications, there are certain things to consider when utilizing cardiovascular signals [34,35]. The ability to accurately and quantitatively determine the meaning of a signal in a short amount of time is essential. This can diagnose various serious cardiovascular diseases, monitor prognosis, and more. The need for entropy methods that employ multiscale techniques has increased due to the inaccurate entropy estimation or invalid calculation of short-length signals with conventional MSE methods [3,4,30]. MSE has certain limitations when it is applied to short-length signals. As the scale factor increases, the length of the coarse-grained time series decreases (original length divided by a scale factor). For short-length signals, this results in extremely short coarse-grained time series at higher scales. Sample Entropy (SampEn) calculation involves counting the occurrences of similar patterns within the time series. Short time series may not contain enough data points to accurately identify and count the recurrence of similar patterns, especially at larger scales. MSE is not defined in this situation, as shown in Figure 7 and Figure 8. In addition, as reported in [36], the coarse-grained time series of MSE is identical to the results of a simple moving average; thus, it may lead to inevitable issues.

In this context, we have shown that the proposed MCRDE can bridge the mismatch between entropy and complexity through simulation using synthetic signals. It shows that the MCRDE values of 1/f are higher than those of WGN over multiple temporal scales. The quantification provided by MCRDE is more consistent than that of MDE because it can tell the difference between 1/f and WGN at all scales, while MDE cannot do that at some scales.

By applying traditional multiscale entropy measures and the proposed MCRDE to the analysis of RR intervals extracted from ECG signals, we aim to differentiate the distinct physiological statuses of subjects. Specifically, it needs to be available for short-length RR intervals as well as for RR intervals with sufficient length. 

In the case of discriminating RR intervals from distinct cardiovascular systems such as CHF, AF, and healthy subjects, MCRDE is more competent than its predecessors for two reasons. First, for short-length RR intervals, MCRDE values are not only valid but also exhibit similar patterns compared to the results of sufficient length. Thus, MCRDE values of different physiological statuses are differentiated regardless of the length of RR intervals. However, MSE suffers from the invalid computation of entropy value in the case of short-length RR intervals, as known previously [31]. The computation of MDE is available for short-length RR intervals, but it cannot discriminate physiological status by quantifying complexity compared to MCRDE.

Following the experiment using young and old subjects’ ECG recordings, similar results from the previous experiment are observed: MCRDE performs better than MSE and MDE irrespective of the length of RR intervals.

Statistical results using the Mann–Whitney U test suggest the following: First, MCRDE is capable of discriminating the complexity between CHF and AF subjects as well as between AF and healthy subjects with short-length RR intervals, while MSE and MDE cannot be computed at high scale factors, and MDE can discriminate different statuses at less scale factors than MCRDE. Second, for longer lengths of RR intervals, MCRDE has a better capacity for discriminating between CHF and healthy subjects. In addition, MDE performs better in discriminating between AF and healthy subjects at small scale factors, while MCRDE shows better performance over higher scale factors. It suggests that MDE and MCRDE can be combined to distinguish between AF and healthy subjects.

Statistical analysis using ECG recordings of healthy subjects shows that MCRDE is a better indicator using short-length and sufficient lengths of RR intervals; thus, MCRDE might play a role in representing subtle changes in cardiovascular signals. 

The early diagnosis of diseases from ECG signals often requires detailed analysis of various cardiac intervals besides the RR interval. These intervals include the QRS duration, PR interval, JT interval, QT interval, and segments like the ST segment [37,38]. For example, the QRS duration is able to indicate bundle branch block or ventricular hypertrophy. In addition, the portion of the ECG between the QRS complex and the T wave is the ST segment. Elevation or depression in the ST segment can indicate myocardial infarction, ischemia, or other forms of heart stress. Thus, the complexity analysis for subtle cardiac intervals would play a pivotal role in providing effective tools for diagnosing cardiac diseases. Beyond the RR interval analysis, it is notable that reflecting correlations between ECG parameters would highlight the emergence of complicated dynamical processes in the cardiovascular system throughout the load by the external stimuli and recovery processes [39].

By analyzing the MCRDE values of ECG signals, it can detect subtle changes that may not be apparent through traditional ECG analysis. Thus, this methodology might play a pivotal role in various clinical applications: early detection of cardiac diseases, monitoring chronic conditions, risk stratification in patients, researching the effects of various drugs or treatments on heart function, and telemedicine and remote monitoring.

Finally, since MCRDE is effective in quantifying dynamic complexity depending on temporal scales, it can be applied to the quantification of other physiological signals such as electroencephalography (EEG), electromyography (EMG), and so on.

## 5. Conclusions

This paper has presented an improved multiscale entropy measure, named MCRDE, by utilizing the cumulative distribution of time series and dispersion entropy in multiple temporal scales. The resultant MCRDE preserves generality and efficient computation of the complexity of underlying cardiovascular signals. Through simulations using synthetic signals and cardiological signals, i.e., RR intervals from ECG signals, the proposed MCRDE is shown to be effective in quantifying and distinguishing different complexity compared to conventional multiscale entropy measures such as MSE and MDE regardless of the length of time series. Based on the advantageous properties of MCRDE, it may provide a promising measure for recognizing various physiological dynamics in biomedical applications. Future works include diverse analyses of ECG signals from other cardiovascular diseases. This work shows that the proposed MCRDE might be a solution for computer-aided diagnosis of cardiovascular diseases.

## Figures and Tables

**Table 1:** Dispersion patterns and the probability of each corresponding dispersion pattern.

z	$z_{1}^{2,3}$	$z_{2}^{2,3}$	$z_{3}^{2,3}$	$z_{4}^{2,3}$	$z_{5}^{2,3}$	$z_{6}^{2,3}$	$z_{7}^{2,3}$	$z_{8}^{2,3}$	$z_{9}^{2,3}$
Dispersion Pattern	{1,1}	{1,2}	{2,2}	{2,2}	{2,3}	{3,2}	{2,2}	{2,3}	{3,1}
π	$\pi_{11}$	$\pi_{12}$	$\pi_{22}$	$\pi_{22}$	$\pi_{23}$	$\pi_{32}$	$\pi_{22}$	$\pi_{23}$	$\pi_{31}$
P	$\frac{1}{9}$	$\frac{1}{9}$	$\frac{3}{9}$	$\frac{3}{9}$	$\frac{2}{9}$	$\frac{1}{9}$	$\frac{3}{9}$	$\frac{2}{9}$	$\frac{1}{9}$

**Table 2:** Statistical analysis results of MSE for RR interval of CHF patients, AF patients, and healthy groups. The shadows indicate that the distinction between the groups is significant. C, A, and H represent CHF, atrial fibrillation, and healthy group, respectively. *s* denotes scale factor and N/A denotes ‘Not Available’.

MSE Statistical Results for RR Interval Time Series									
s	*p*-Value								
	*N* = 100			*N* = 500			*N* = 1000		
	C–A	C–H	A–H	C–A	C–H	A–H	C–A	C–H	A–H
1	0.0629	0.8843	0.7870	0.1478	0.0134	0.9172	0.0203	9.50 × 10^−4^	0.9834
2	0.6625	0.0483	0.1904	0.0849	0.0011	0.2891	0.0409	8.5507 × 10^−5^	0.1006
3	0.6961	0.0396	0.1515	0.0366	2.7538 × 10^−4^	0.0773	0.0409	2.4494 × 10^−5^	0.0358
4	0.6295	0.0150	0.0188	0.0695	1.6847 × 10^−4^	0.0210	0.1237	6.4704 × 10^−6^	0.0235
5	0.9817	N/A	N/A	0.0508	5.1825 × 10^−4^	0.0235	0.1478	2.9429 × 10^−5^	0.0083
6	0.6961	N/A	N/A	0.3232	0.0020	0.0210	0.2603	1.2042 × 10^−4^	0.0150
7	N/A	N/A	0.0415	0.7652	0.0057	0.0483	0.3953	2.3416 × 10^−4^	0.0150
8	N/A	N/A	N/A	0.8722	0.0106	0.0586	0.3232	1.6847 × 10^−4^	0.0210
9	0.3559	N/A	N/A	0.9817	0.0065	0.0150	0.6259	6.0454 × 10^−4^	0.0261
10	N/A	N/A	N/A	0.9451	0.0323	0.1095	0.5972	0.0026	0.0643
11	N/A	N/A	N/A	0.9817	0.0532	0.0845	0.5351	0.0030	0.0483
12	N/A	N/A	N/A	0.6625	0.0438	0.0323	0.6625	0.0034	0.0235
13	N/A	N/A	N/A	0.6961	0.2706	0.1904	0.8005	0.0044	0.0438
14	N/A	N/A	N/A	0.5053	0.3286	0.1292	0.9996	0.0261	0.0643
15	N/A	N/A	N/A	0.7652	0.1904	0.0706	0.9817	0.0134	0.0586
16	N/A	N/A	N/A	0.4763	0.3714	0.0923	0.6625	0.0119	0.0168
17	N/A	N/A	N/A	0.2064	0.8516	0.1095	0.5351	0.0773	0.0483
18	N/A	N/A	N/A	0.2234	0.7552	0.0845	0.6259	0.0923	0.0532
19	N/A	N/A	N/A	0.2802	0.7238	0.1637	0.3953	0.2530	0.0586
20	N/A	N/A	N/A	0.0935	0.9834	0.1292	0.3462	0.4176	0.0845
21	N/A	N/A	N/A	0.0508	0.9834	0.0438	0.3953	0.2891	0.0483
22	N/A	N/A	N/A	0.1354	0.9834	0.0586	0.1753	0.5747	0.0050
23	N/A	N/A	N/A	0.0981	0.4176	0.1767	0.1753	0.6625	0.0261
24	N/A	N/A	N/A	0.0768	0.7238	0.2361	0.3703	0.9834	0.4176
25	N/A	N/A	N/A	0.1237	0.6623	0.2048	0.1904	0.9668	0.0532

**Table 3:** Statistical analysis results of MDE for RR interval of CHF patients, AF patients, and healthy groups. The shadows indicate that the distinction between the groups is significant. C, A, and H represent CHF, atrial fibrillation, and healthy group, respectively. In addition, s denotes scale factor and N/A denotes ‘Not Available’.

MDE Statistical Results for RR Interval Time Series									
s	*p*-Value								
	*N* = 100			*N* = 500			*N* = 1000		
	C–A	C–H	A–H	C–A	C–H	A–H	C–A	C–H	A–H
1	0.0769	0.4176	0.3496	0.0159	0.0013	0.7552	0.0123	0.0013	0.5747
2	0.6458	0.4797	0.9172	0.0291	9.502 × 10^−4^	0.1637	0.0229	5.182 × 10^−4^	0.0706
3	0.9817	0.0111	0.0093	0.0366	5.183 × 10^−4^	0.0586	0.0123	4.227 × 10^−5^	0.0119
4	0.7823	0.015	0.0036	0.018	2.035 × 10^−5^	0.0119	0.0366	1.398 × 10^−5^	0.0323
5	0.8528	0.1067	0.1943	0.0849	0.0011	0.002	0.0508	5.054 × 10^−5^	0.0034
6	0.4095	0.2408	0.8754	0.0628	1.016 × 10^−4^	0.0034	0.0456	1.688 × 10^−5^	0.0039
7	0.7041	0.0025	0.0022	0.2234	0.003	0.0034	0.1129	3.53 × 10^−5^	0.0323
8	0.5386	0.0074	0.0429	0.0935	0.0016	0.0396	0.1611	4.227 × 10^−5^	0.0065
9	0.8363	0.0627	0.0504	0.2802	0.003	0.0323	0.1753	2.943 × 10^−5^	0.0083
10	0.6573	0.4473	0.19	0.5972	0.0773	0.2201	0.1904	0.0013	0.0738
11	1	0.8111	0.8111	0.9451	0.0291	0.1006	0.2603	3.79 × 10^−4^	0.0188
12	0.5983	0.0112	0.0047	0.8005	0.00437	0.3496	0.2802	8.551 × 10^−5^	0.0261
13	0.7301	0.2443	0.4723	0.9633	0.0068	0.022	0.4347	0.0015	0.0773
14	0.5774	0.1361	0.3162	0.2061	0.4174	0.0613	0.5053	0.0034	0.0483
15	0.6528	0.1361	0.0602	0.6289	0.2699	0.2887	0.5052	0.002	0.0807
16	0.3531	N/A	0.3162	0.3939	0.9171	0.2041	0.4621	0.002	0.0234
17	0.3531	N/A	0.3162	0.1174	0.8351	0.0842	0.7652	0.015	0.1141
18	N/A	N/A	N/A	0.357	1	0.2605	0.6792	0.0358	0.1291
19	N/A	N/A	N/A	0.4207	0.2879	0.0501	0.9085	0.0199	0.0248
20	N/A	N/A	N/A	0.3207	0.4124	0.0645	0.5657	0.1005	0.0188
21	N/A	N/A	N/A	0.0874	0.9665	0.0301	0.5657	0.1833	0.0323
22	N/A	N/A	N/A	0.0058	0.5951	0.0105	0.3701	0.1901	0.0806
23	N/A	N/A	N/A	0.0517	0.4599	0.1034	0.4096	0.4045	0.0321
24	N/A	N/A	N/A	0.2481	0.8996	0.2099	0.5351	0.5746	0.0844
25	N/A	N/A	N/A	0.0357	0.4385	0.0799	0.2602	0.7869	0.1094

**Table 4:** Statistical analysis results of MCRDE for RR interval of CHF patients, AF patients, and healthy groups. The shadows indicate that the distinction between the groups is significant. C, A, and H represent CHF, atrial fibrillation, and healthy group, respectively. In addition, s denotes scale factor and N/A denotes ‘Not Available’.

MCRDE Statistical Results for RR Interval Time Series									
s	*p*-Value								
	*N* = 100			*N* = 500			*N* = 1000		
	C–A	C–H	A–H	C–A	C–H	A–H	C–A	C–H	A–H
1	0.1237	0.6625	0.1006	0.6625	0.3941	0.3496	0.3703	0.0396	0.0643
2	0.0628	0.5194	0.0706	0.1611	0.0773	0.7238	0.1354	0.0073	0.1292
3	0.0628	0.2361	0.1292	0.1237	0.0235	0.4928	0.1129	0.0044	0.1292
4	0.0159	0.2706	0.0706	0.1753	0.0483	0.4176	0.1129	0.0039	0.0706
5	0.0203	0.1292	0.0643	0.1029	0.0065	0.2706	0.0695	0.0026	0.0845
6	0.0366	0.3496	0.0586	0.1029	0.0106	0.3496	0.0628	0.0015	0.0643
7	0.018	0.6326	0.0261	0.0695	0.0057	0.2201	0.0409	0.0013	0.0532
8	0.0258	0.3286	0.1006	0.0366	0.0044	0.3286	0.0366	0.0015	0.119
9	0.0123	0.5194	0.0323	0.0935	0.0083	0.3496	0.0508	0.0015	0.0643
10	0.0108	0.6929	0.0291	0.0508	0.0020	0.2048	0.0291	4.44 × 10^−4^	0.0358
11	0.0094	0.3085	0.021	0.0456	0.0039	0.3085	0.0409	8.186 × 10^−4^	0.0586
12	0.0041	0.4669	0.0044	0.0565	0.0020	0.1904	0.0409	5.183 × 10^−4^	0.0358
13	0.018	0.6326	0.0358	0.0769	0.0026	0.1904	0.0456	8.186 × 10^−4^	0.0532
14	0.0229	0.9172	0.0134	0.0508	0.0017	0.2361	0.0456	6.045 × 10^−4^	0.0235
15	0.0456	0.7552	0.0073	0.0203	0.0026	0.1515	0.0229	7.04 × 10^−4^	0.0261
16	0.168	0.9834	0.1006	0.0291	0.0020	0.2201	0.0366	6.045 × 10^−4^	0.0643
17	0.1129	0.4928	0.0323	0.0456	0.0020	0.2048	0.0326	4.44 × 10^−4^	0.015
18	0.1351	0.4927	0.0199	0.0769	0.0083	0.3941	0.0258	5.18 × 10^−4^	0.0358
19	0.1029	0.9172	0.0438	0.0565	0.0039	0.1190	0.0366	4.44 × 10^−4^	0.0291
20	0.1353	0.7552	0.0807	0.0203	0.0013	0.2361	0.0229	6.045 × 10^−4^	0.0261
21	0.0384	0.4293	0.0047	0.0769	0.0057	0.4928	0.0258	2.754 × 10^−4^	0.0188
22	0.2061	0.1764	0.0306	0.0326	7.04 × 10^−4^	0.2361	0.0258	4.436 × 10^−4^	0.0358
23	0.3118	0.1048	0.0159	0.0203	0.0026	0.4669	0.0203	3.233 × 10^−4^	0.0106
24	0.818	0.2614	0.1291	0.0229	0.0022	0.2891	0.0159	4.44 × 10^−4^	0.0065
25	0.5962	0.8352	0.2526	0.0229	0.0022	0.3714	0.0326	1.988 × 10^−4^	0.0073

**Table 5:** Statistical analysis results for RR interval of healthy young and elderly groups. The shadows indicate that the distinction between the groups is significant. In addition, s denotes scale factor and N/A denotes ‘Not Available’.

Statistical Results for RR Interval Time Series of Healthy Young and Elderly Groups									
s	*p*-Value								
	MSE			MDE			MCRDE		
	*N* = 100	*N* = 500	*N* = 1000	*N* = 100	*N* = 500	*N* = 1000	*N* = 100	*N* = 500	*N* = 1000
1	3.0786 × 10^−4^	5.7598 × 10^−4^	5.7598 × 10^−4^	0.0016	5.7598 × 10^−4^	4.9369 × 10^−4^	0.0042	0.0028	0.0028
2	0.009	0.0021	0.0019	0.2051	2.6217 × 10^−4^	3.6093 × 10^−4^	0.0016	1.3564 × 10^−4^	0.0011
3	0.0202	0.0062	0.0032	0.1131	0.0251	0.0021	0.0019	1.1457 × 10^−4^	3.6093 × 10^−4^
4	0.0744	0.0128	0.0062	0.7675	0.2628	0.229	0.0037	4.8063 × 10^−5^	4.2247 × 10^−4^
5	0.068	0.0144	0.0090	0.7451	0.6187	0.4807	0.089	2.329 × 10^−5^	1.6033 × 10^−4^
6	0.2625	0.0512	0.0128	0.0057	0.3951	0.3837	0.0028	1.1457 × 10^−4^	7.8021 × 10^−4^
7	0.3835	0.0465	0.0181	0.3614	0.0971	0.1711	0.0021	4.02 × 10^−5^	9.0585 × 10^−4^
8	N/A	0.1150	0.0381	0.3554	0.0649	0.1150	0.0344	3.3568 × 10^−5^	0.0014
9	0.1896	0.0971	0.0225	0.1146	0.0152	0.2133	0.0062	1.0922 × 10^−5^	0.0037
10	0.3919	0.1249	0.0564	1	0.0619	0.0251	0.0564	4.8063 × 10^−5^	7.8021 × 10^−4^
11	N/A	0.1466	0.0564	0.5765	0.0709	0.0021	0.0279	3.0786 × 10^−4^	6.709 × 10^−4^
12	N/A	0.1354	0.0680	1	0.0889	0.0101	0.0465	5.7371 × 10^−5^	0.0032
13	N/A	0.2544	0.0512	N/N	0.0036	0.0191	0.7400	3.3568 × 10^−5^	0.0014
14	N/A	0.1466	0.0890	0.3506	0.3093	0.0744	0.4551	0.0011	0.0032
15	N/A	0.3837	0.1150	0.3506	0.0039	0.0042	0.3192	0.0016	0.0079
16	N/A	0.1585	0.1466	0.3506	0.0326	0.002	0.5614	4.9369 × 10^−4^	0.0016
17	N/A	0.1985	0.0971	N/A	0.0050	0.0048	0.0360	1.1457 × 10^−4^	0.0025
18	N/A	0.2290	0.0421	N/A	0.0170	0.0181	0.5457	6.709 × 10^−4^	0.0016
19	N/A	0.3615	0.1249	N/A	0.0323	0.0237	0.0529	9.0585 × 10^−4^	0.0037
20	N/A	0.1585	0.0815	N/A	0.0770	0.0399	0.0881	6.709 × 10^−4^	0.0062
21	N/A	0.8357	0.0971	N/A	0.0317	0.0512	N/A	0.0011	0.0048
22	N/A	0.2290	0.1249	N/A	0.1895	0.0037	N/A	9.0585 × 10^−4^	0.0128
23	N/A	0.1585	0.0680	N/A	0.0267	0.0026	N/A	3.0786 × 10^−4^	0.0019
24	N/A	0.4428	0.1249	N/A	0.0414	0.0101	N/A	0.0037	0.0114
25	N/A	0.2455	0.1249	N/A	0.1171	0.0034	N/A	0.0016	0.0114

## Data Availability

The ECG dataset used for this study is publicly available on https://www.physionet.org/ (accessed on 15 November 2023).
